# Interactive effects between lead-cadmium co-exposure and VEGFA gene polymorphisms on renal dysfunction: a gene–environment interaction study

**DOI:** 10.3389/fpubh.2025.1627634

**Published:** 2025-09-25

**Authors:** Yaotang Deng, Yunman Wen, Weixia Duan, Zhiqiang Zhao, Guoliang Li, Jiazhen Zhou, Le Yang, Jieyi Yang, Yapei Sun, Manyi Qiu, Lili Liu

**Affiliations:** ^1^Chongqing Key Laboratory of Prevention and Treatment for Occupational Diseases and Poisoning, The First Affiliated Hospital of Chongqing Medical and Pharmaceutical College, Chongqing, China; ^2^Department of Toxicology, Guangdong Province Hospital for Occupational Disease Prevention and Treatment, Guangzhou, China

**Keywords:** lead, cadmium, polymorphism, VEGFA, gene–environment interaction, renal dysfunction

## Abstract

**Background:**

Lead (Pb) and cadmium (Cd) are common persistent environmental pollutants, may cause renal dysfunction following long-term exposure. This study investigated whether vascular endothelial growth factor a (VEGFA) gene polymorphisms modify the association between Pb and Cd exposure with renal dysfunction risk, given the key role of gene–environment interactions in kidney pathogenesis.

**Methods:**

A cross-sectional study of 408 workers was undertaken from a Pb-Cd smelter in Guangdong Province, China in 2023. Metals in blood and urine were measured using Inductively coupled plasma-Mass Spectrometry (ICP-MS). Additive, dominant and recessive genetic models were employed to analyze differences in genotype distribution of rs3025010, rs10434 and rs833061 between normal and renal dysfunction groups. Interaction analyses were conducted to examine the combined effects of blood lead (BPb) and urinary cadmium (UCd) exposure with these polymorphisms under different genetic models on renal dysfunction risk.

**Results:**

For rs833061 locus, BPb showed statistically significant differences in both the additive and recessive models (*p* < 0.05), while renal function exhibited differences in the additive and dominant models (*p* < 0.05). For rs3025010, BPb showed significant differences in the recessive model (*p* = 0.05), and renal function demonstrated differences in both additive and dominant models (*p* < 0.05). Multivariate regression analysis identified BPb and UCd as risk factors for renal dysfunction, with odds ratios ranging from 1.40 to 3.46 (*p* < 0.05). Interaction analyses revealed interactions between rs3025010 and Pb [BPb × rs3025010: OR (95%CI) = 0.69(0.49, 0.88)] in dominant model. The rs10434 locus interactions with Pb and Cd in both the additive [OR (95%CI) = 0.60 (0.31, 0.91)] and recessive models [OR (95%CI) = 0.51 (0.27, 0.85)].

**Conclusion:**

This study identified significant gene–environment interactions between VEGFA polymorphisms (rs3025010 and rs10434) and Pb-Cd co-exposure in renal dysfunction. These findings suggest that screening for these polymorphisms could identify high-risk populations for targeted prevention and control strategies.

## 1 Introduction

The kidneys are vital detoxification organs responsible for the eventual excretion of most harmful substances from the body (1, 2). However, early-stage kidney injury is relatively insidious. Once detected, it is often irreversible (3). This characteristic underscores the critical importance of identifying high-risk populations and implementing early diagnostic strategies for renal dysfunction (4). Both Cd and Pb are heavy metals widely utilized in various industrial settings (5, 6). These metals possess significant industrial applications, however, prolonged and excessive exposure to these metals poses substantial health risks, with the kidneys being particularly sensitive target organs (7, 8). These heavy metals are recognized as important etiological factors in nephrotoxicity, as chronic exposure can progressively compromise renal dysfunction, potentially culminating in renal dysfunction, and long-term exposure increases the risk of chronic renal complications (9–11).

Exposure levels for Pb and Cd are categorized based on toxicological reference points, with low-dose Pb exposure defined as blood levels below the No Observed Adverse Effect Level (NOAEL) of approximately 50–100 μg/L, and low-dose Cd exposure as urinary concentrations below the NOAEL of 1–2 μg/gCr, while high-dose exposures exceed the Lowest Observed Adverse Effect Level (LOAEL) of 100–150 μg/L for Pb and 2–5 μg/gCr for Cd (12, 13). Both metals exhibit distinct toxicokinetic profiles that contribute to their nephrotoxic potential: Pb is primarily absorbed through the gastrointestinal tract (5–15% in adults) and respiratory system (30–50%), subsequently binding to erythrocytes and distributing to soft tissues including the kidneys, where it accumulates in proximal tubular cells and disrupts cellular function through oxidative stress, mitochondrial dysfunction, and interference with essential metal enzyme systems (7, 14). Cd, absorbed mainly via inhalation (10–50%) and ingestion (3–5%), forms complexes with metallothionein in the liver before redistribution to the kidneys, where it preferentially accumulates in proximal tubular cells with an extraordinarily long biological half-life of 10–30 years compared to Pb′s 1–2 month half-life in soft tissues (15, 16). Both metals induce nephrotoxicity through shared mechanisms including reactive oxygen species generation, lipid peroxidation, DNA damage, and apoptotic pathway activation, while Cd additionally causes specific tubular dysfunction through metallothionein-cadmium complex-mediated lysosomal damage and disruption of calcium homeostasis (17, 18). The clearance of these metals occurs predominantly through renal excretion, creating a paradoxical situation where the primary elimination organ becomes the target of toxicity, with renal clearance rates of approximately 0.6–2.0 mL/min for Pb and 0.1–0.5 mL/min for Cd (19, 20). Regarding renal recovery, proximal tubular epithelial cells demonstrate regenerative capacity within 7–14 days following acute injury through dedifferentiation and proliferation of surviving cells, however, chronic exposure to Pb and Cd can overwhelm this regenerative capacity, leading to progressive fibrosis and irreversible functional decline (21, 22).

Long-term low-dose exposure to Pb and Cd can lead to renal dysfunction in some individuals, while others remain unaffected (23, 24). This intriguing phenomenon may be closely related to environmental response genes, which play a crucial role in responding to environmental pollutants. These genes regulate the body’s response mechanisms to environmental stressors, and genetic variations within them can modulate sensitivity to toxic substances. Gene–environment interaction refers to the combined effects of genetic and environmental factors in influencing disease occurrence and progression (25, 26). It accounts for the fact that among individuals exposed to the same environmental factors, some remain disease-free whereas others are more prone to illness. Single-nucleotide polymorphisms (SNPs), which represent the most common form of genetic variation, are of great significance in gene–environment interaction. By influencing the expression of genes in response to environmental exposures, SNPs affect an individual’s health status (27, 28). Chen et al. (29) has shown that plasma myeloperoxidase interacts with metals, contributing to chronic kidney disease, which further supports this point of view. Environmental response genes are crucial for identifying high-risk populations, particularly those who exhibit different health responses under similar environmental exposures, providing a basis for developing personalized prevention and treatment strategies.

As a crucial environmental response gene, VEGFA plays a key role in responding to oxidative stress and tissue damage (30). Studies have shown that the VEGFA gene is associated with the development of various diseases, including kidney and cardiovascular diseases (31). In the kidneys, VEGFA serves as an important angiogenic factor primarily secreted by podocytes and tubular epithelial cells (32, 33), where it plays a critical role in both physiological and pathological processes. In particularly, changes in the expression and secretion of VEGFA significantly impact both functional recovery and the progression of damage (34). The absence of VEGFA can result in ischemic injury to the renal vasculature and tissue (34). Specific SNPs in the VEGFA gene may relate to the risk of renal dysfunction among workers with long-term exposure to Pb and Cd.

In this study, we investigated the interactions between Cd and Pb exposure and VEGFA SNPs on renal dysfunction, which can help identify high-risk populations sensitive to Cd and Pb, and facilitate early prevention and intervention.

## 2 Methods

### 2.1 Study population

As previously mentioned, this cross-sectional study enrolled occupationally exposed workers from a Pb-Cd smelter in Guangdong Province, China from January 2023 to December 2023 (35). Eligible participants met the following inclusion criteria: (1) minimum employment duration of 1 year in metal processing operations; (2) medication-free status for ≥2 weeks preceding biological sampling; (3) absence of major chronic pathologies including cardiovascular, hepatorenal, gastrointestinal, autoimmune disorders, or malignancies. During routine occupational health surveillance, fasting biological specimens (8-h overnight fast) were systematically collected through venipuncture and mid-stream urine sampling. Initial clinical assessments encompassed urinalysis and complete blood count. Residual samples were cryopreserved in aliquots at −80 °C for subsequent molecular analyses. The study protocol received ethical approval from the Institutional Review Board of Guangdong Provincial Hospital for Occupational Disease Prevention and Control (GDHOD-IRB-2023-018), with written informed consent obtained from all participants prior to enrollment.

### 2.2 Exposure assessment for Cd and Pb

Analytical determination of UCd and BPb concentrations was performed using ICP-MS (7500ce, Agilent Technologies, United States) with minor modification of the method as previously described (36). Briefly, 100 μL samples were first diluted quantitatively (10-fold) with 1% (v/v) HNO_3_ and then the mixed solution and carefully aspirated into ICP-MS for determination. Each sample was measured in triplicate to obtain an average value (μg/L). For quality control purposes, the Multi-element Solution (CLMS-2 N, SPEX CertiPrep, United States) was utilized to confirm that the values of Cd and Pb measured in the reference samples were within the recommended range. The intra-day and inter-day coefficient variation were 12.5 and 6.5%. Urinary creatinine levels were used to normalize UCd concentrations. All measurements exceeded the instrument-specific detection limits (0.15 μg/L for urinary Cd; 0.08 μg/L for blood Pb), ensuring complete dataset usability.

### 2.3 Renal function assessment and outcome ascertainment

Blood creatinine was measured with a clinical chemistry autoanalyzer. Estimated Glomerular Filtration Rate (eGFR, mL/min/1.73m^2^) was computed using age, sex, and serum creatinine, based on the equations from Chronic Kidney Disease Epidemiology Collaboration (CKD-EPI) (37). Participants with sustained eGFR < 90 mL/min/1.73m^2^ across two consecutive measurements (≥3-month interval) were classified as having renal dysfunction (38).

### 2.4 Selection and genotyping of SNPs

Three SNPs in VEGFA were selected using the HapMap database and the Haploview 4.2 software (Broad Institute, Cambridge, MA, United States). The SNP rs833061 resides in the 5′-untranslated region (UTR) promoter domain of VEGFA, rs3025010 resides in the Exon 2 non-synonymous mutation domain and rs10434 resides in the 3’-UTR miRNA binding domain. The minor allele frequencies of these three SNPs were greater than 5%, and the linkage disequilibrium *r*^2^ > 0.8. Subsequently, genotyping of these SNP loci across various regions of VEGFA gene was performed using next-generation sequencing technology.

### 2.5 Covariates

Demographic information (age and gender), lifestyles (smoking, and alcohol drinking status), and occupational history (working position and years, shift work) were collected by questionnaire interviews. Smoking status was defined as smoking ≥1 cigarette per day for at least 6 months, and alcohol consumption was defined as drinking ≥1 time per month at least 6 months. Participants not meeting these criteria were categorized as non-smokers or non-drinkers, respectively. Self-reported chronic disease history was cross-verified with medical records for accuracy. Anthropometric measurements (height in meters and weight in kilograms) were obtained using an automated device, and body mass index (BMI) was calculated as weight divided by height squared (kg/m^2^).

### 2.6 Statistical analysis

Categorical variables were presented as numbers (%), and continuous variables were presented as medians (interquartile range, IQR). The SNP genotypes of VEGFA were evaluated using the *χ*^2^ test or Fisher’s exact test for categorical variables, and Student’s *t*-test for categorical variables. The *Z* for Kruskal–Wallis test and *χ*^2^ for chi-square test or Fisher’s exact text were calculated. The criteria for statistical significance was set at *p* < 0.05 with a confidence interval of 95%.

Interaction effects were estimated using an interaction model in the ‘epiR’ package (39), with corresponding OR and 95% confidence intervals (95% CIs). We included interaction terms composed of metals and SNPs separately into the regression model, treating the marginal effects of the interaction terms as the interaction effects.

All statistical analyses were performed by R software (version 4.31, R Foundation for Statistical Computing). A two-sided *p*-value of <0.05 was considered statistically significant.

## 3 Results

### 3.1 Characteristics of study populations

The general characteristics, concentrations of BPb and UCd, and renal function indicators of all participants are summarized in Table 1. This study comprised 408 subjects who were stratified into normal (*n* = 260) and renal dysfunction (*n* = 148) groups based on eGFR. It revealed significant between-group differences in gender distribution [*χ*^2^_(1, 408)_ = 11.82, Cramer *V* = 0.16, *p* < 0.001], smoking status [*χ*^2^_(1, 408)_ = 16.54, Cramer *V* = 0.20, *p* < 0.001], and shift work status [*χ*^2^_(1, 408)_ = 5.31, Cramer *V* = 0.10, *p* < 0.05]. The UCd concentrations were significantly elevated in the renal dysfunction group (median 3.68 μg/gCr) compared to normal group [3.08 μg/gCr; *Z*_(1, 408)_ = −3.30, Epsilon-squared = 0.13, *p* < 0.001]. It was paralleled by BPb levels, with substantially higher concentrations in the renal dysfunction group (median 115.89 μg/L) versus the normal group [70.21 μg/L; *Z*_(1, 408)_ = −15.11, Epsilon-squared = 0.56, *p* < 0.001]. CREA was significantly elevated in the renal dysfunction group [median 76.75 μmol/L versus 72.00 μmol/L; *Z*_(1, 408)_ = −3.32, Epsilon-squared = 0.13, *p* < 0.001], while eGFR were correspondingly reduced [median 95.35 versus 102.26; *Z*_(1, 408)_ = −2.95, Epsilon-squared = 0.12, *p* = 0.003]. As a biomarker of kidney injury, the concentration of β_2_-microglobulin (β_2_-MG) levels exhibited marked elevation in the renal dysfunction group (median 74.76 μg/gCr) compared to normal subjects [62.86 μg/gCr; *Z*_(1, 408)_ = −5.84, Epsilon-squared = 0.08, *p* < 0.001], further substantiating the differential renal function profiles between groups.

**Table 1:** Baseline characteristics (*n* = 409).

Variables	Total (*n* = 408)	Normal group (*n* = 260)	Renal dysfunction group (*n* = 148)	Effect size	*p*-value
Age	45.00 (42.00, 47.00)	45.00 (43.00, 47.00)	45.00 (42.00, 47.00)	0.001	0.53
Working years	25.00 (22.00, 26.00)	25.00 (22.00, 26.00)	25.00 (23.00, 26.00)	<0.001	0.62
Gender				0.16	<0.001^***^
Male	355 (87.0)	215 (82.7)	140 (94.6)		
Female	53 (13.0)	45 (17.3)	8 (5.4)		
Smoking				0.20	<0.001^***^
No	167 (40.9)	87 (33.5)	80 (54.1)		
Yes	241 (59.1)	173 (66.5)	68 (45.9)		
Alcohol drinking				0.04	0.20
No	307 (75.2)	201 (77.3)	106 (71.6)		
Yes	101 (24.8)	59 (22.7)	42 (28.4)		
Shift work				0.10	0.02^*^
Yes	313 (76.7)	190 (73.1)	123 (83.1)		
No	95 (23.3)	70 (26.9)	25 (16.9)		
BMI (kg/m^2^)	22.77 (21.26, 24.21)	22.77 (21.21, 24.10)	22.77 (21.50, 24.22)	<0.001	0.65
Cd (μg/gCr)	3.32 (2.35, 5.14)	3.08 (2.26, 4.76)	3.68 (2.59, 5.67)	0.13	<0.001^***^
Pb (μg/L)	87.14 (57.23, 107.52)	70.21 (37.02, 86.33)	115.89 (105.18, 144.42)	0.56	<0.001^***^
CREA (μmol/L)	73.00 (57.63, 84.80)	72.00 (52.77, 83.17)	76.75 (66.62, 87.28)	0.13	<0.001^***^
eGFR (mL/min/1.73m^2^)	99.97 (84.11, 122.87)	102.26 (85.40, 131.15)	95.35 (80.87, 112.20)	0.12	0.003^**^
β_2_-MG (μg/gCr)	70.39 (54.27, 82.64)	62.86 (47.84, 74.42)	74.76 (59.91, 89.45)	0.08	<0.001^***^

Quantitative data were expressed as median (IQR) deviation, which were compared using the Kruskal–Wallis test. Categorical data were expressed in the form of number (%). The difference of frequencies among different groups was tested by the *χ*^2^ test or Fisher’s exact text, with statistical significance indicated as follows: *p < 0.05, **p < 0.01, ***p < 0.001.

Effect size: Epsilon-squared for Kruskal–Wallis test and Cramer *V* for *χ*^2^ test or Fisher’s exact text.

BMI, body mass index; CREA, Creatinine; UREA, blood urea nitrogen; eGFR, estimated glomerular filtration rate.

### 3.2 Distribution of metal exposure level, and renal function in various genetic models

The distribution of demographic characteristics, metal exposure levels, and renal function across different genetic models of VEGFA rs833061 polymorphism is presented in Table 2. In the additive model, participants were categorized as TT (*n* = 216), CT (*n* = 159), and CC (*n* = 33) genotypes. For the dominant model, subjects were classified as TT (*n* = 216) and CT + CC (*n* = 192) groups, while in the recessive model, they were stratified into CT + TT (*n* = 375) and CC (*n* = 33) groups. In both the additive model and recessive model, UCd were not comparable among genotype groups in all genetic models (*p* > 0.05), while BPb showed statistically significant differences. Notably, the distribution of renal function status showed significant differences across genotypes in the additive model [*χ*^2^_(2, 408)_ = 8.06, Cramer *V* = 0.12, *p* < 0.05] and the dominant model [*χ*^2^_(2, 408)_ = 7.59, Cramer *V* = 0.13, *p* < 0.05], but not in the recessive model [*χ*^2^_(2, 408)_ = 2.32, Cramer *V* = 0.06, *p* = 0.13]. In the additive model, the proportion of renal dysfunction was higher in CT (42.14%) and CC (48.48%) genotypes compared to TT (30.09%). Similarly, in the dominant model, subjects with CT + CC genotypes exhibited a higher prevalence of renal dysfunction (43.23%) than those with the TT genotype (30.09%), suggesting potential associations between VEGFA rs833061 polymorphism and renal function.

**Table 2:** Distribution of metal exposure levels and renal function across different genetic models of VEGFA rs833061 locus.

Variables	Additive model				Dominant model			Recessive model		
	TT (*n* = 216)	CT (*n* = 159)	CC (*n* = 33)	*p-*value (Effect size)	TT (*n* = 216)	CT + CC (*n* = 192)	*p-*value (Effect size)	CT + TT (*n* = 375)	CC (*n* = 33)	*p-*value (Effect size)
Age (yrs)	45.00 (42.00, 47.00)	45.00 (43.00, 47.00)	46.00 (42.00, 48.00)	0.72 (0.002)	45.00 (42.00, 47.00)	45.00 (42.00, 47.00)	0.62 (<0.001)	45.00 (42.00, 47.00)	46.00 (42.00, 48.00)	0.65 (<0.001)
Working years (year)	25.00 (23.00, 26.00)	25.00 (22.00, 26.00)	25.00 (22.00, 26.00)	0.91 (<0.001)	25.00 (23.00, 26.00)	25.00 (22.00, 26.00)	0.83 (<0.001)	25.00 (23.00, 26.00)	25.00 (22.00, 26.00)	0.67 (<0.001)
Gender (*n*, %)				0.63 (<0.001)			0.39 (<0.001)			0.49 (<0.001)
Male	186 (85.7)	140 (88.1)	30 (90.9)		185 (85.7)	170 (88.5)		325 (86.7)	30 (90.9)	
Female	31 (14.3)	19 (11.9)	3 (9.1)		31 (14.3)	22 (11.5)		50 (13.3)	3 (9.1)	
Smoking (*n*, %)				0.41 (<0.001)			0.93 (<0.001)			0.20 (0.04)
Ever smoking	89 (41.0)	62 (39.0)	17 (51.5)		88 (41.0)	79 (41.1)		150 (40.0)	17 (51.5)	
Never smoking	128 (59.0)	97 (61.0)	16 (48.5)		128 (59.0)	113 (58.9)		225 (60.0)	16 (48.5)	
Alcohol drinking (*n*, %)				0.51 (<0.001)			0.74 (<0.001)			0.23 (0.03)
Ever drinking	53 (24.4)	38 (23.9)	11 (33.3)		52 (24.4)	49 (25.5)		90 (24.0)	11 (33.3)	
Never drinking	164 (75.6)	121 (76.1)	22 (66.7)		164 (75.6)	143 (74.5)		285 (76.0)	22 (66.7)	
Shift work (*n*, %)				0.94 (<0.001)			0.95 (<0.001)			0.77 (<0.001)
Yes	166 (76.9)	121 (76.1)	26 (78.8)		166 (76.9)	147 (76.6)		287 (76.5)	26 (78.8)	
No	50 (23.1)	38 (23.9)	7 (21.2)		50 (23.1)	45 (23.4)		88 (23.5)	7 (21.2)	
BMI (kg/m^2^)	22.77 (21.26, 24.09)	22.77 (21.26, 24.22)	23.03 (21.71, 24.22)	0.63 (0.002)	22.77 (21.26, 24.09)	22.77 (21.44, 24.22)	0.51 (0.001)	22.77 (21.26, 24.15)	23.03 (21.71, 24.22)	0.38 (0.002)
UCd (μg/gCr)	3.22 (2.36,5.05)	3.42 (2.37,5.21)	3.58 (2.24,5.28)	0.77 (0.001)	3.22(2.36, 5.05)	3.48(2.26, 5.26)	0.65 (<0.001)	3.29 (2.36, 5.09)	3.58 (2.24, 5.28)	0.51 (0.001)
BPb (μg/L)	134.51 (105.67,152.67)	135.96 (103.60,158.78)	153.37 (133.33,180.26)	0.02^*^ (0.13)	134.51 (105.67, 152.67)	138.79 (113.47, 160.86)	0.08 (0.008)	135.18 (105.67, 155.40)	153.37 (133.33, 180.26)	0.01^**^ (0.14)
Renal function				0.02^*^ (0.12)			0.01^**^ (0.13)			0.13 (0.06)
Normal	151 (69.91)	92 (57.86)	17 (51.52)		151 (69.91)	109 (56.77)		243 (64.80)	17 (51.52)	
Dysfunction	65 (30.09)	67 (42.14)	16 (48.48)		65 (30.09)	83 (43.23)		132 (35.20)	16 (48.48)	

Quantitative data were expressed as median (IQR) deviation, which were compared using the Kruskal–Wallis test. Categorical data were expressed in the form of number (%). The difference of frequencies among different groups was tested by the χ^2^ test or Fisher’s exact text, with statistical significance indicated as follows: *p < 0.05, **p < 0.01.

Effect size: Epsilon-squared for Kruskal–Wallis test and Cramer *V* for *χ*^2^ test or Fisher’s exact text.

BMI, body mass index; CREA, Creatinine; UREA, blood urea nitrogen; eGFR, estimated glomerular filtration rate.

Table 3 presents the distribution of different genetic models at the rs3025010 locus. For the additive model, participants were categorized as TT (*n* = 205), CT (*n* = 165), and CC (*n* = 38) genotypes. In the dominant model, subjects were classified as TT (*n* = 205) versus CT + CC (*n* = 203) groups, while the recessive model stratified participants into CT + TT (*n* = 370) and CC (*n* = 38) groups. In the recessive model, BPb showed a statistically significant difference [*Z*_(1, 408)_ = −1.96, Epsilon-squared = 0.009, *p* = 0.05], with higher concentrations observed in the CC genotype group (153.42 μg/L) compared to the CT + TT group (135.68 μg/L). Regarding renal function status, significant differences were observed in both additive [χ^2^_(2, 408)_ = 7.66, Cramer *V* = 0.12, *p* = 0.02] and dominant [*χ*^2^_(1, 408)_ = 6.48, Cramer *V* = 0.12, *p* = 0.01] models. In the additive model, the proportion of renal dysfunction was notably higher in CT (40.61%) and CC (50.00%) genotypes compared to TT (30.24%). Similarly, in the dominant model, subjects with CT + CC genotypes exhibited a higher prevalence of renal dysfunction (42.36%) than those with the TT genotype (30.24%). However, no significant difference was detected in the recessive model [*χ*^2^_(1, 408)_ = 3.41, Cramer *V* = 0.08, *p* = 0.06], although there was a trend toward higher dysfunction rates in the CC group. These findings suggest potential associations between VEGFA rs3025010 polymorphism and renal function, particularly when considering Pb exposure.

**Table 3:** Distribution of metal exposure levels and renal function across different genetic models of VEGFA rs3025010 locus.

Variables	Additive model				Dominant model			Recessive model		
	TT (*n* = 205)	CT (*n* = 165)	CC (*n* = 38)	*p*-value (Effect size)	TT (*n* = 205)	CT + CC (*n* = 203)	*p*-value (Effect size)	CT + TT (*n* = 370)	CC (*n* = 38)	*p*-value (Effect size)
Age (yrs)	45.00 (42.00, 47.00)	44.00 (42.00, 47.00)	46.00 (43.00, 48.00)	0.08 (0.01)	45.00 (42.00, 47.00)	45.00 (42.00, 47.00)	0.23 (0.004)	45.00 (42.00, 47.00)	46.00 (43.00, 48.00)	0.16 (0.005)
Working years (year)	25.00 (23.00, 26.00)	25.00 (22.00, 26.00)	25.00 (23.25, 26.00)	0.38 (0.005)	25.00 (23.00, 26.00)	25.00 (22.00, 26.00)	0.73 (0.003)	25.00 (22.00, 26.00)	25.00 (23.25, 26.00)	0.25 (0.003)
Gender (*n*, %)				0.57 (<0.001)			0.49 (<0.001)			0.47 (<0.001)
Male	176 (85.8)	144 (87.3)	35 (92.1)		176 (85.8)	179 (88.2)		320 (86.5)	35 (92.1)	
Female	29 (14.2)	21 (12.7)	3 (7.9)		29 (14.2)	24 (11.8)		50 (13.5)	3 (7.9)	
Smoking (*n*, %)				0.30 (0.03)			0.70 (<0.001)			0.12 (0.06)
Ever smoking	82 (40.0)	65 (39.34)	20 (52.6)		82 (40.0)	85 (41.9)		147 (39.7)	20 (52.6)	
Never smoking	123 (60.0)	100 (60.6)	18 (47.4)		123 (60.0)	118 (58.1)		223 (60.3)	18 (47.4)	
Alcohol drinking (*n*, %)				0.19 (0.06)			0.12 (0.06)			0.16 (0.05)
Ever drinking	44 (21.5)	44 (26.7)	13 (34.2)		44 (21.5)	57 (28.1)		88 (23.8)	13 (34.2)	
Never drinking	161 (78.5)	121 (73.3)	25 (65.8)		161 (78.5)	146 (71.9)		282 (76.2)	25 (65.8)	
Shift work (*n*, %)				0.74 (<0.001)			0.95 (<0.001)			0.46 (<0.001)
Yes	157 (76.6)	125 (75.8)	31 (81.6)		157 (76.6)	156 (76.9)		282 (76.2)	31 (81.6)	
No	48 (23.4)	40 (24.2)	7 (18.4)		48 (23.4)	47 (23.1)		88 (23.8)	7 (18.4)	
BMI (kg/m^2^)	22.76 (21.22, 24.09)	22.68 (21.38,24.16)	23.76 (21.68,25.29)	0.18 (0.008)	22.76 (21.22, 24.09)	22.84 (21.52, 24.22)	0.37 (0.002)	22.74 (21.26, 24.09)	23.76 (21.68, 25.29)	0.07 (0.008)
UCd (μg/gCr)	3.23 (2.41,5.01)	3.35 (2.20,5.34)	3.63 (2.60,4.88)	0.57 (0.003)	3.23 (2.41, 5.01)	3.46 (2.26, 5.32)	0.63 (<0.001)	3.26 (2.35, 5.17)	3.63 (2.60, 4.88)	0.29 (0.003)
BPb (μg/L)	133.98 (107.74, 153.33)	138.35 (105.67,158.45)	153.42 (115.55,172.14)	0.10 (0.01)	133.98 (107.74, 153.33)	138.82 (108.78, 160.48)	0.15 (0.005)	135.68 (106.19, 155.18)	153.42 (115.55, 172.14)	0.05^*^ (0.009)
Renal function				0.02^*^ (0.12)			0.01^**^ (0.12)			0.06 (0.08)
Normal	143 (69.76)	98 (59.39)	19 (50.00)		143 (69.76)	117 (57.64)		241 (65.14)	19 (50.00)	
Dysfunction	62 (30.24)	67 (40.61)	19 (50.00)		62 (30.24)	86 (42.36)		129 (34.86)	19 (50.00)	

Quantitative data were expressed as median (IQR) deviation, which were compared using the Kruskal–Wallis test. Categorical data were expressed in the form of number (%). The difference of frequencies among different groups was tested by the *χ*^2^ test or Fisher’s exact text, with statistical significance indicated as follows: *p < 0.05, **p < 0.01.

Effect size: Epsilon-squared for Kruskal–Wallis test and Cramer *V* for *χ*^2^ test or Fisher’s exact text.

BMI, body mass index; CREA, Creatinine; UREA, blood urea nitrogen; eGFR, estimated glomerular filtration rate.

As for rs10434 locus, participants were classified as GG (*n* = 224), AG (*n* = 165), and AA (*n* = 19) genotypes in additive model. In the dominant model, subjects were categorized as GG (*n* = 224) versus AG + AA (*n* = 184), while the recessive model stratified participants into AG + GG (*n* = 389) and AA (*n* = 19) groups (Table 4). There were no statistically significant differences in UCd and BPb levels among all genotypes across all genetic models [Additive model: (*Z*_(2, 408)_ = 1.72, Epsilon-squared = 0.004, *p* = 0.42), Dominant model: (*Z*_(1, 408)_ = −0.10, Epsilon-squared < 0.001, *p* = 0.92), Recessive model: (*Z*_(1, 408)_ = −1.24, Epsilon-squared = 0.004, *p* = 0.21)]. Renal function revealed no statistically significant differences across genotypes in any of the genetic models [Additive model: (*χ*^2^_(2, 408)_ = 1.96, Cramer *V* < 0.001, *p* = 0.38), Dominant model: (*χ*^2^_(1, 408)_ = 1.95, Cramer *V* = 0.5, *p* = 0.16), Recessive model: (*χ*^2^_(1, 408)_ = 0.19, Cramer *V* < 0.001, *p* = 0.66)]. These findings suggest that VEGFA rs10434 polymorphism alone may not significantly influence renal function in this study population, though further investigation considering potential gene–environment interactions may be warranted.

**Table 4:** Distribution of metal exposure levels and renal function across different genetic models of VEGFA rs10434 locus.

Variables	Additive model				Dominant model			Recessive model		
	GG (*n* = 224)	AG (*n* = 165)	AA (*n* = 19)	*p*-value (Effect size)	GG (*n* = 224)	AG + AA (*n* = 184)	*p*-value (Effect size)	AG + GG (*n* = 389)	AA (*n* = 19)	*p*-value (Effect size)
Age (yrs)	45.00 (42.00, 47.00)	44.00 (42.00, 47.00)	47.00 (44.50, 49.00)	0.05^*^ (0.01)	45.00 (42.00, 47.00)	45.00 (42.00, 47.00)	0.54 (<0.001)	45.00 (42.00, 27.00)	47.00 (44.00, 49.00)	0.03^*^ (0.01)
Working years (year)	25.00 (23.00, 26.00)	25.00 (22.00, 26.00)	24.00 (23.00, 25.00)	0.52 (0.003)	25.00 (23.00, 26.00)	25.00 (22.00, 26.00)	0.50 (0.001)	25.00 (22.00, 26.00)	24.00 (23.00, 25.00)	0.29 (0.003)
Gender (*n*, %)				0.29 (0.03)			0.39 (<0.001)			0.47 (0.02)
Male	192 (85.7)	148 (89.7)	15 (78.9)		192 (85.7)	163 (88.6)		340 (87.4)	15 (78.9)	
Female	32 (14.3)	17 (10.3)	4 (21.1)		32 (14.3)	21 (11.4)		49 (12.6)	4 (21.1)	
Smoking (*n*, %)				0.99 (<0.001)			0.89 (<0.001)			0.92 (<0.001)
Ever smoking	91 (40.6)	68 (41.2)	8 (42.1)		91 (40.6)	76 (41.3)		159 (40.9)	8 (42.1)	
Never smoking	133 (59.4)	97 (58.8)	11 (57.9)		133 (59.4)	108 (58.7)		230 (59.1)	11 (57.9)	
Alcohol drinking (*n*, %)				0.97 (<0.001)			0.90 (<0.001)			1.00 (<0.001)
Ever drinking	56 (25.0)	40 (24.2)	5 (26.3)		56 (25.0)	45 (24.5)		96 (24.7)	5 (26.3)	
Never drinking	168 (75.0)	125 (75.8)	14 (73.7)		168 (75.0)	139 (75.5)		293 (75.3)	14 (73.7)	
Shift work (*n*, %)				0.71 (<0.001)			0.50 (<0.001)			0.97 (<0.001)
Yes	169 (75.4)	130 (78.8)	14 (73.7)		55 (24.5)	40 (21.7)		299 (76.9)	14 (73.7)	
No	55 (24.6)	35 (21.2)	5 (26.3)		169 (75.5)	144 (78.3)		90 (23.1)	5 (26.3)	
BMI (kg/m^2^)	22.67 (21.25, 24.09)	22.77 (21.26, 24.22)	23.99 (21.77, 24.22)	0.47 (0.003)	22.67 (21.25, 24.09)	22.86 (21.29, 24.22)	0.71 (<0.001)	22.76 (21.26, 24.17)	23.99 (21.77, 24.22)	0.30 (0.003)
UCd (μg/gCr)	3.31 (2.37, 5.25)	3.31 (2.34, 4.95)	4.29 (2.59, 7.03)	0.42 (0.004)	3.31 (2.37, 5.25)	3.33 (2.35, 5.04)	0.92 (<0.001)	3.31 (2.35, 5.08)	4.29 (2.59, 7.03)	0.21 (0.004)
BPb (μg/L)	138.79 (112.95, 161.28)	132.61 (99.46, 153.33)	137.29 (118.28, 149.22)	0.17 (0.009)	138.79 (112.95, 161.28)	131.43 (103.08, 153.17)	0.05^*^ (0.008)	136.98 (105.67, 157.66)	137.29 (118.28, 149.22)	0.95 (<0.001)
Renal function				0.38 (<0.001)			0.16 (0.05)			0.66 (<0.001)
Normal	136 (60.71)	111 (67.27)	13 (68.42)		136 (60.71)	124 (67.39)		247 (63.50)	13 (68.42)	
Dysfunction	88 (39.29)	54 (32.73)	6 (31.58)		88 (39.29)	60 (32.61)		142 (36.50)	6 (31.58)	

Quantitative data were expressed as median (IQR) deviation, which were compared using the Kruskal–Wallis test. Categorical data were expressed in the form of number (%). The difference of frequencies among different groups was tested by the *χ*^2^ test or Fisher’s exact text, with statistical significance indicated as follows: *p < 0.05.

Effect size: Epsilon-squared for Kruskal–Wallis test and Cramer *V* for *χ*^2^ test or Fisher’s exact text.

BMI, body mass index; CREA, Creatinine; UREA, blood urea nitrogen; eGFR, estimated glomerular filtration rate.

### 3.3 Interaction effects between metal exposure and SNPs in renal function

**Figure 1:**
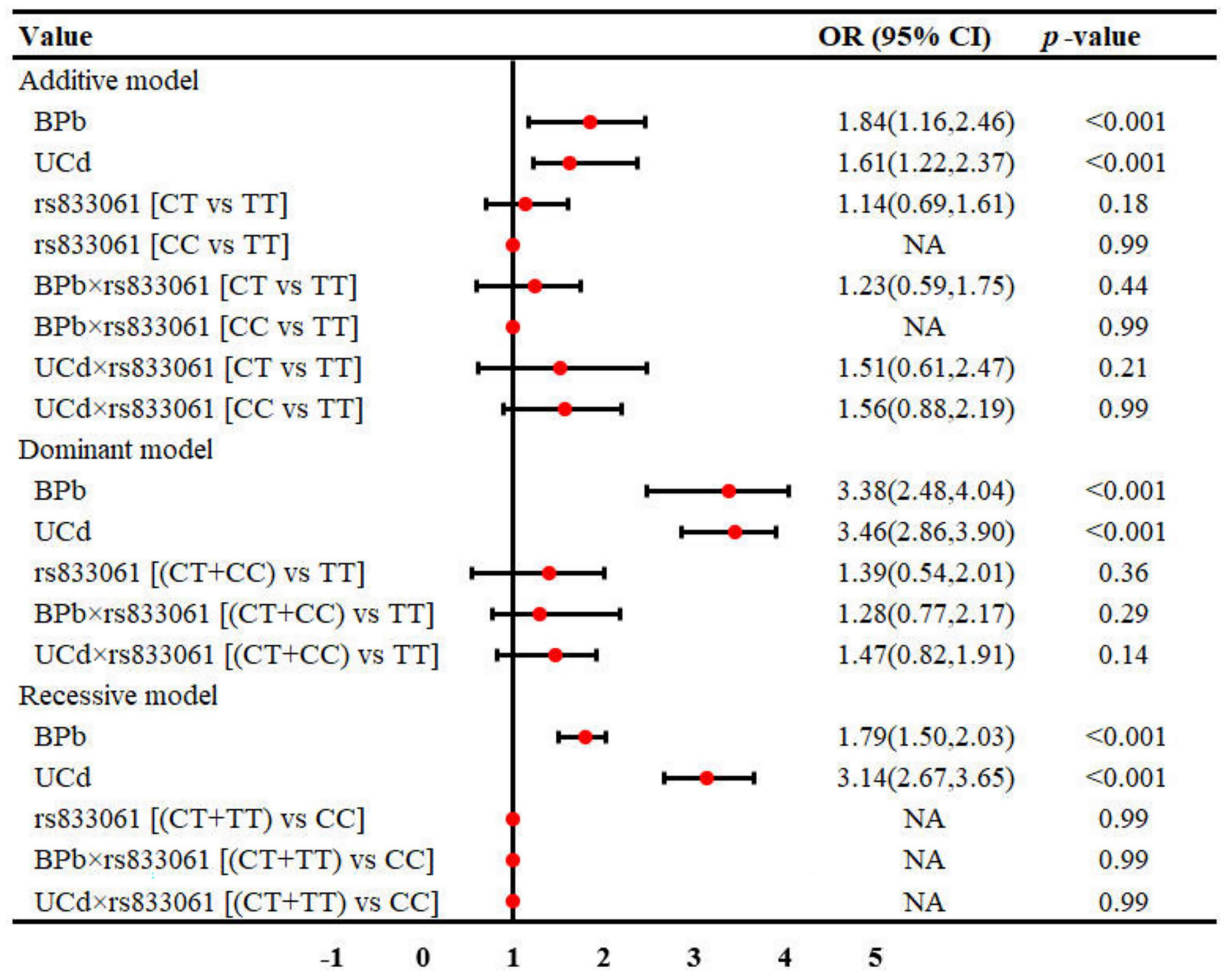
Interaction effects between metal exposure (UCd and BPb) and rs833061 in renal function. The interaction effects were adjusted for age, gender, smoking, drinking and shift work status, and BMI.

**Figure 2:**
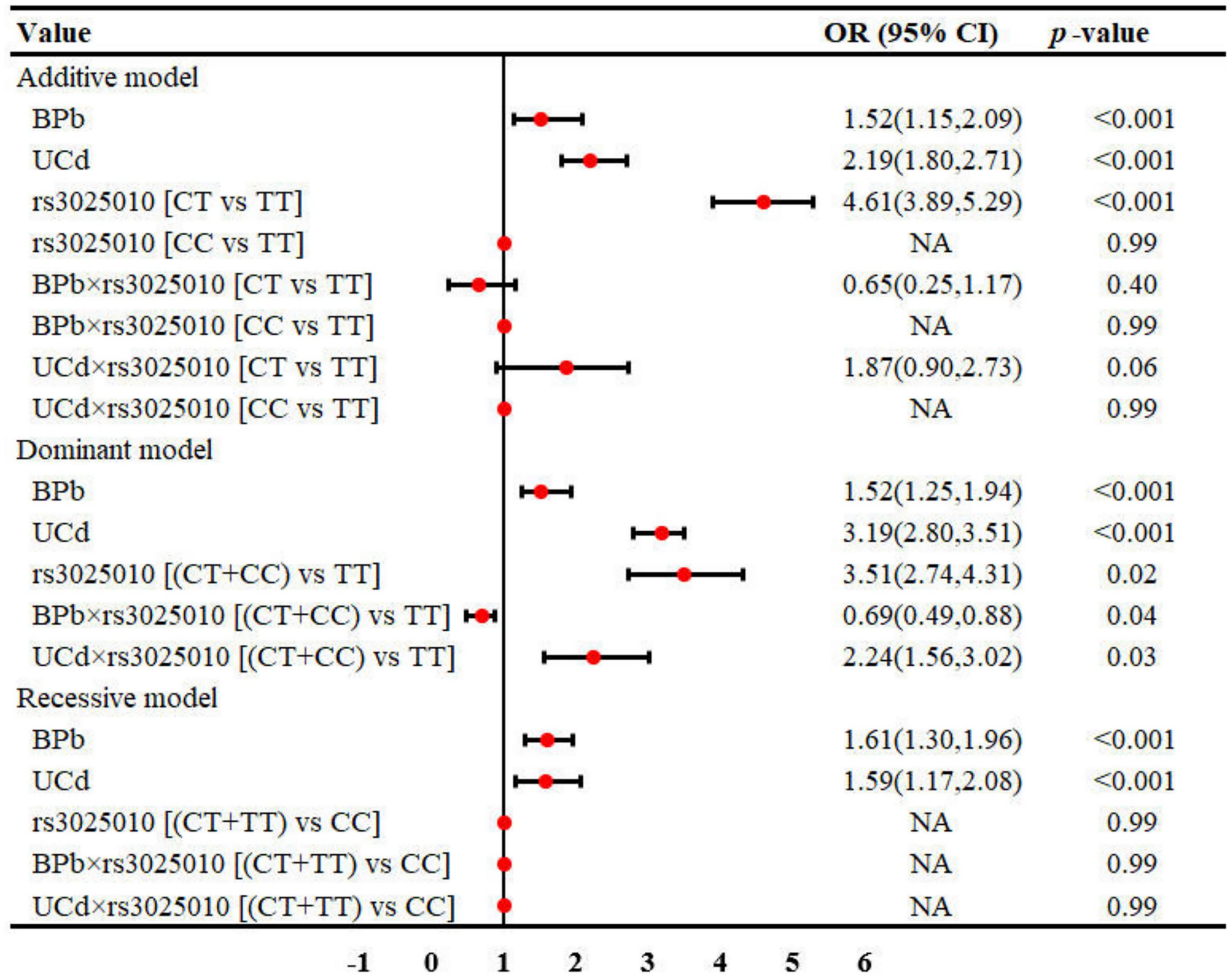
Interaction effects between metal exposure (UCd and BPb) and rs3025010 in renal function. The interaction effects were adjusted for age, gender, smoking, drinking and shift work status, and BMI.

**Figure 3:**
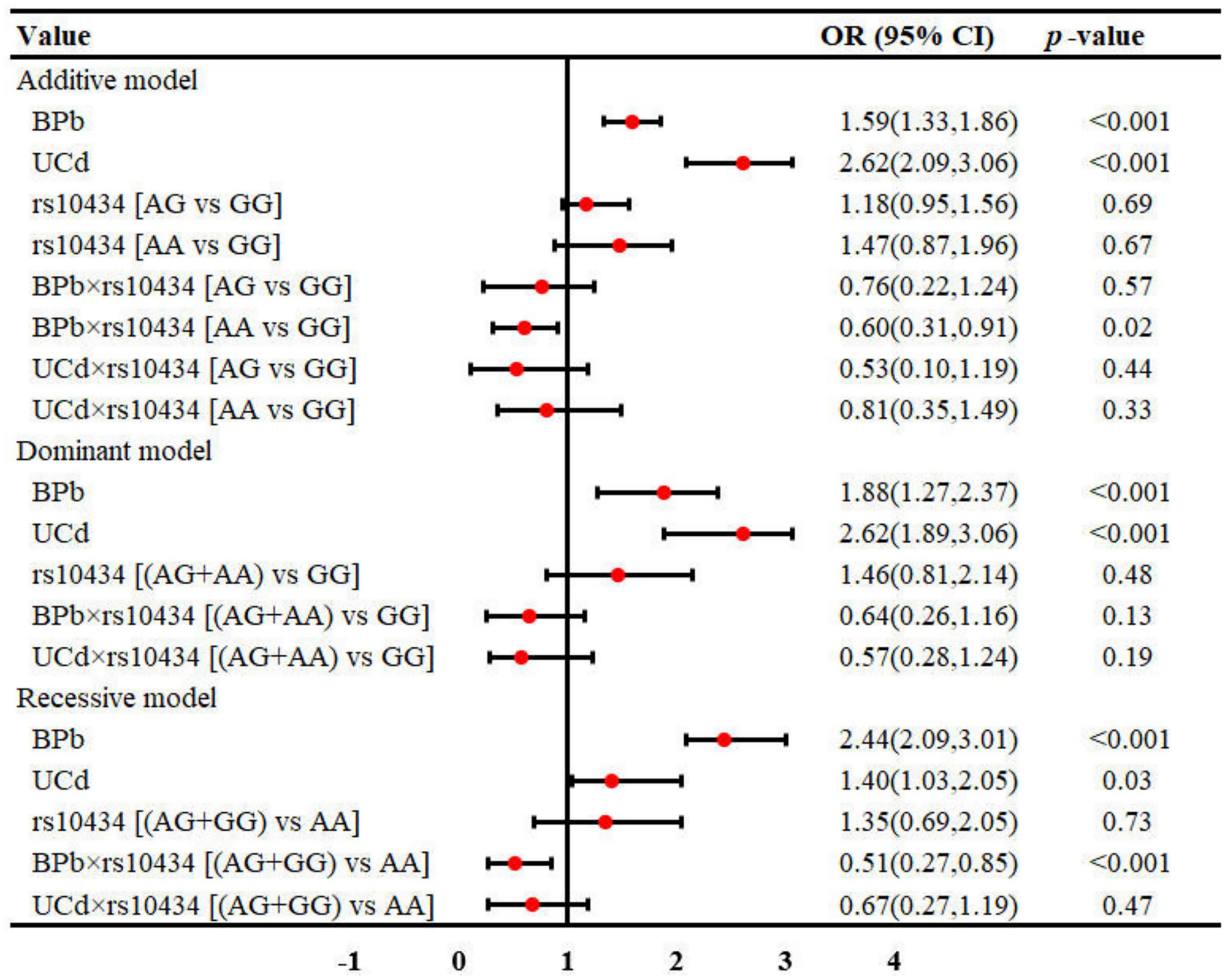
Interaction effects between metal exposure (UCd and BPb) and rs10434 in renal function. The interaction effects were adjusted for age, gender, smoking, drinking and shift work status, and BMI.

The interaction between metal exposure and SNPs on renal function under three genetic models was shown in Figures 1–3. Both BPb and UCd showed positive associations with renal dysfunction across the three genetic models in these polymorphic loci, with ORs ranging from 1.40 to 3.46. For rs3025010, there was a positive interaction with BPb [OR (95% CI) = 0.69 (0.49, 0.88), *p* = 0.04], while a negative interaction with UCd [OR (95% CI) = 2.24 (1.56, 3.02), *p* = 0.03] in the dominant model. For rs3025010, the CT + CC group showed elevated risk of renal dysfunction compared to the wild type [OR (95% CI) = 3.51 (2.74, 4.31), *p* = 0.04] in the dominant model. Furthermore, interaction analysis revealed that for the rs10434 locus, there was an interaction between BPb and rs10434 [OR (95% CI) = 0.60 (0.31, 0.91), *p* = 0.02] in the additive model, while in the recessive model, the AG + GG group showed a negative association compared to the AA group [OR (95% CI) = 0.51 (0.27, 0.85), *p* < 0.001]. These findings suggest that high-risk populations for lead and cadmium exposure can be identified through rs3025010 and rs10434 polymorphisms.

## 4 Discussion

Renal dysfunction represents an early manifestation of chronic kidney injury which is often insidious and irreversible, making screening of high-risk populations extremely important. In this study, we identified associations between VEGFA gene polymorphisms at loci rs833061, rs3025010, and rs10434 and renal dysfunction. Notably, our study identified distinct interactive effects between Pb exposure and the rs3025010, rs10434 loci, as well as between Cd exposure and the rs10434 locus, in modulating renal dysfunction. These gene–environment interactions suggest that genotyping at these loci could enhance early identification of populations at elevated risk for Pb- and Cd-induced renal impairment.

As a crucial factor in maintaining the integrity of the glomerular filtration barrier and promoting peritubular capillary angiogenesis, VEGFA is closely related to renal function (40, 41). Under normal physiological conditions, VEGFA regulates vascular permeability and neovascularization through paracrine and autocrine mechanisms (42). However, aberrant VEGFA expression may exacerbate renal injury through multiple pathways under conditions of Pb and Cd exposure (43–45). Specifically, Pb and Cd activate oxidative stress and the TGF-β1/Smad3 signaling pathway, upregulating VEGFA expression, which promotes peritubular capillary proliferation but simultaneously induces fibroblast activation (46, 47), resulting in extracellular matrix deposition and interstitial fibrosis. Furthermore, overexpression of VEGFA can aggravate inflammatory responses by activating the PI3K/AKT/mTOR pathway, accelerating glomerulosclerosis and renal function decline (48).

Genetic polymorphisms regulate gene function by modulating transcription and expression levels, thereby influencing physiological processes and disease susceptibility (49, 50). In our study, we identified significant associations between renal function and two polymorphic loci within the VEGFA gene: rs10434 and rs3025010. The rs10434 polymorphism is located within the 3’-UTR region of the VEGFA gene, a critical regulatory site known to influence mRNA stability, translation efficiency, and subcellular localization (51, 52). However, no prior studies have investigated the association between this locus and renal function. Our findings revealed a novel gene–environment interaction, suggesting that the mutation at rs10434 may synergize with lead exposure to exert a protective effect on renal dysfunction. This observation highlights the potential role of rs10434 in modifying the nephrotoxic consequences of environmental heavy metal exposure through its regulatory functions in VEGFA expression. Regarding rs3025010, this polymorphism resides within the Exon 2 non-synonymous mutation domain, where genetic alterations may induce non-synonymous mutations that modify protein folding patterns and structural stability (53). Garrigos et al. (54) identified this locus as a prognostic and predictive biomarker for renal cell carcinoma, likely related to its role in VEGFA-mediated abnormal angiogenesis. Collectively, our findings suggest that polymorphisms at rs10434 and rs3025010 loci are associated with Pb and Cd-mediated renal dysfunction, potentially through mechanisms that affect VEGFA transcription, translation, protein conformation, and stability, thereby altering renal sensitivity to heavy metal exposure. Furthermore, genotyping these loci could facilitate the identification of susceptible populations for targeted early preventive interventions (55).

In contrast to traditional view that examines genetic and environmental factors independently, most diseases arise from the intricate interplay between these two determinants. For example, interactions between TIMP3, Pb exposure, and renal function that may contribute to the development of chronic kidney disease (56). The complex relationship between environmental exposures and genetic susceptibility in disease pathogenesis has gained increasing attention recently. Our investigation revealed that the rs3025010 and rs10434 polymorphism exhibited heightened vulnerability to renal dysfunction induced by Pb and Cd exposure, and the rs10434 variant appeared to mitigate this risk and provide nephroprotective effects. Both Pb and Cd represent significant heavy metal contaminants not only in industrial metal smelting operations but also as persistent environmental pollutants (57). Chronic exposure to these elements can induce nephrotoxicity with potentially irreversible structural and functional consequences. Previous studies have documented interactions between these heavy metals and specific genetic polymorphisms affecting renal function. Chia et al. (56) identified significant interactions between six polymorphic loci on the ALAD gene and Pb exposure that collectively influenced renal function biomarkers. Furthermore, the rs28366003 polymorphism located in the promoter region of the MT2A gene has been demonstrated to modulate Cd accumulation in renal tissues (58), further substantiating the combined influence of genetic variants and heavy metal exposure on kidney health. The identification of gene–environment interactions provides a compelling mechanistic explanation for the observed heterogeneity in health outcomes among individuals with comparable exposure levels. This has profound implications for public health practice, facilitating the development of personalized health management strategies tailored to individual genetic profiles and environmental exposure patterns.

While this study was conducted in a Chinese population, the identified gene–environment interactions between VEGFA polymorphisms and heavy metal exposure have significant implications for global public health. In India, where both Pb and Cd contamination represent major environmental health challenges, particularly in industrial regions such as West Bengal and Rajasthan (59), our findings could inform targeted screening strategies. The high prevalence of chronic kidney disease in India, affecting approximately 17% of the adult population (60), combined with widespread heavy metal exposure, makes genotype-guided risk assessment particularly relevant. In United States and Europe, where environmental heavy metal exposure occurs primarily through occupational settings and contaminated sites, these genetic markers could serve as valuable tools for personalized occupational health monitoring. The Occupational Safety and Health Administration (OSHA) guidelines for lead exposure monitoring could potentially incorporate genetic screening to identify workers at elevated risk (61).

The practical applications of our findings extend beyond traditional metal smelting operations to encompass a broader spectrum of occupational settings characterized by heavy metal exposure. Electronic waste recycling represents a rapidly expanding industry where workers face significant exposure to both lead and cadmium through the dismantling of electronic components (62). Battery manufacturing and recycling industries, particularly those involved in lead-acid and nickel-cadmium battery processing, represent another critical application domain. Workers in these industries experience chronic exposure to both metals, making genetic screening for VEGFA polymorphisms potentially valuable for risk stratification (63). Similarly, workers in pigment and paint manufacturing, particularly those producing cadmium-based pigments, could benefit from genetic susceptibility assessment to guide occupational health practices.

This study made several meaningful contributions. Primarily, it explored the interactions between kidney-damaging heavy metals Pb and Cd and genetic factors, which enables the screening of high-risk populations for precision management. Additionally, identifying more SNP loci that interact with Pb and Cd could better explain the mechanisms of renal damage induced by these heavy metals. However, several limitations of this study should be acknowledged. First, this is a cross-sectional study, which precludes causal inference. In future research, we will follow this cohort to investigate the interactions between the aforementioned SNP loci and Pb and Cd exposure, as well as their causal relationships with renal dysfunction. Second, the sample size needs to be further expanded to ensure the stability of recessive models in interaction analyses.

## 5 Conclusion

Our study identified rs3025010 and rs10434 polymorphisms in the VEGFA gene that may be associated with renal dysfunction induced by Pb and Cd exposure. Furthermore, both SNP loci demonstrated significant interactions with Pb and Cd, highlighting the complex mechanisms between environmental factors and genetic polymorphisms. Through these findings, high-risk populations for Pb and Cd exposure can be identified, enabling the development of precise prevention strategies.

## Data Availability

The original contributions presented in the study are included in the article/supplementary material, further inquiries can be directed to the corresponding author/s.
